# Tooth loss is associated with increased risk of esophageal cancer: evidence from a meta-analysis with dose-response analysis

**DOI:** 10.1038/srep18900

**Published:** 2016-01-08

**Authors:** Qi-Lin Chen, Xian-Tao Zeng, Zhi-Xiao Luo, Xiao-Li Duan, Jie Qin, Wei-Dong Leng

**Affiliations:** 1 Department of Stomatology, Taihe Hospital, Hubei University of Medicine, Shiyan 442000, Hubei Province, China; 2 Center for Evidence-Based and Translational Medicine, Department of Stomatology, Zhongnan Hospital of Wuhan University, Wuhan 430071, P.R. China; 3 Department of Digestive Medicine, Taihe Hospital, Hubei University of Medicine, Shiyan, 442000, P.R. China

## Abstract

Epidemiological studies have revealed the association between tooth loss and the risk of esophageal cancer (EC); however, consistent results were not obtained from different single studies. Therefore, we conducted the present meta-analysis to evaluate the association between tooth loss and EC. We conducted electronic searches of PubMed until to February 10, 2015 to identify relevant observational studies that examined the association between tooth loss and the risk of EC. Study selection and data extraction from eligible studies were independently performed by two authors. The meta-analysis was conducted using Stata 12.0 software. Finally eight eligible publications with ten studies involving 3 cohort studies, 5 case-control studies, and 1 cross-sectional study were yielded. Meta-analysis identified tooth loss increased risk of EC 1.30 times (Relative risk = 1.30, 95% confidence interval = 1.06–1.60, *I*^2^ = 13.5%). Dose-response analysis showed linear relationship between tooth loss and risk of EC (RR = 1.01, 95%CI = 1.00–1.03; *P* for non-linearity test was 0.45). Subgroup analysis proved similar results and publication bias was not detected. In conclusion, tooth loss could be considered to be a significant and dependent risk factor for EC based on the current evidence.

Tooth loss is known to considerably influence food choice, diet, nutrition intake, and esthetics^1^. It has also has been considered to impact oral health-related quality of life^2^, aggravate people with severe mental illnesses^3^, and increase the risk of cardiovascular disease^4^,^5^,^6^ as well as head and neck cancer (HNC)^7^. Zheng *et al.* (1990)^8^ first reported that tooth loss is a strong risk factor for oral cancer, and this association was further confirmed by Zeng *et al.* (2013) using meta-analysis^7^. For head and neck is adjoined to esophagus; besides, tooth loss as well as HNC and esophageal cancer (EC) share common risk factors, including age, gender, diabetes, social and geographical disparities, smoking, and alcohol consumption^9^,^10^,^11^,^12^,^13^,^14^,^15^,^16^. Therefore, the real relationship between tooth loss and EC need to be elucidated.

Abnet *et al.* (2001) reported that tooth loss increased the risk of developing esophageal squamous cell carcinoma (ESCC) in China^17^. Following this, many relevant epidemiological studies have been published; however, these studies have provided inconsistent or even contradicting results. The present study aimed to systematically review existing literature and to analyze the relationship between the tooth loss and the risk of EC using a meta-analysis. We hypothesize that tooth loss is associated with an increased risk of EC.

## Methods

This meta-analysis was performed according to the Preferred Reporting Items for Systematic Reviews and Meta-Analyses (PRISMA) statement^18^.

### Eligibility criteria

Cohort, case-control, and cross-sectional studies that evaluated the association between tooth loss and EC while meeting the following criteria were considered eligible for inclusion: (1) full-text articles could be obtained; (2) clear diagnostic criteria for EC and definition of tooth loss were reported; and (3) either the adjusted and/or unadjusted hazard ratios (HRs), odds ratios (ORs), or relative risks (RRs) and their corresponding 95% confidence intervals (CIs) or the numbers of events that could be used for their calculation were reported. When studies with overlapping data were eligible, we chose the one with the most comprehensive information. Two authors independently evaluated the eligibility of all the retrieved studies, and disagreements were resolved by discussion.

### Search strategy

For identifying relevant studies, we conducted electronic searches of the PubMed database until February 10, 2015 using the search terms (esophageal OR oesophageal OR gullet) AND (dentition OR “tooth loss” OR edentulous OR “lost of tooth”). Reference lists of recent reviews and the selected papers and were manually screened to identify additional relevant studies and avoid erroneous exclusions. Only publications in English were included.

### Data extraction

Two authors independently extracted the following information from each eligible study: last name of the first author; year of publication; study design; country of origin; sample size; age; pathological characteristics of EC; unadjusted or adjusted ORs, RRs, and HRs and relevant 95% CIs or standard errors (SEs); and the covariates for the adjusted point estimates.

### Data analysis

Statistical analysis was performed using STATA 12.0 software. First, we transformed ORs, RRs, or HRs and their CIs to their natural logarithms and SEs. We directly considered HR as RR^7^,^19^ and computed the combined RRs and 95% CIs from the estimates reported in each study^20^,^21^. Heterogeneity was quantified using *I*^2^ values and chi-square test^22^; when both *I*^2^ ≤ 50% and *p* > 1.0 indicated no or acceptable heterogeneity^23^, we used the fixed-effects model; otherwise, we used the random-effects model. In addition, we performed subgroup analyses on the basis of stratified ORs, RRs, and HRs, given that these pooled may result in the overestimation of OR variance^24^. We also conducted a dose-response meta-analysis using STATA 12.0 software with restricted cubic spline function by the method of Orini^25^ for those studies reported sufficient data, including RRs, serving size, and the sample size in each categories. Furthermore, we performed subgroups analyses on the basis of the study design, and type of cancer, adjustment, and definition of reference group. Publication bias was assessed by visual inspection of the funnel plots^26^.

## Results

### Study selection and characteristics

**Figure 1:**
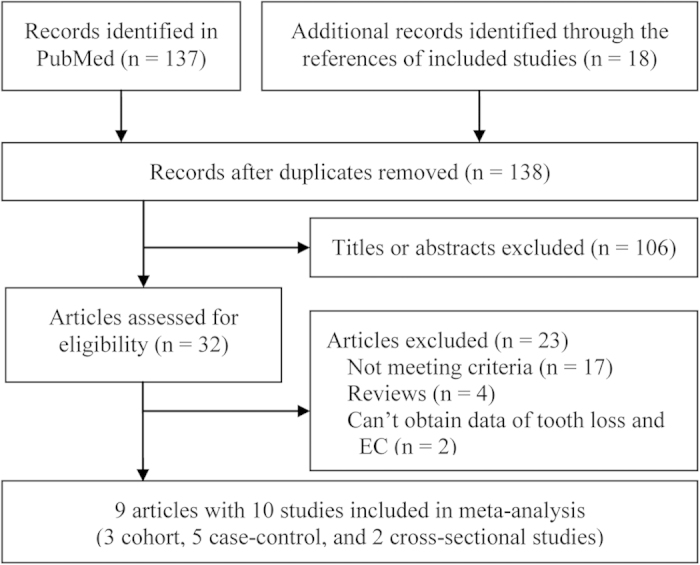
Flow chart from identification of eligible studies to final inclusion. EC, esophageal cancer.

From the 155 records initially identified, 8 articles involving 3 cohort studies^17^,^27^,^28^, 5 case-control studies^29^,^30^,^31^,^32^, and 1 cross-sectional studies^33^ were selected for the present meta-analysis. A detailed flow chart of the selection process is shown in Fig. 1.

Among the selected studies, the study of Guha *et al.*^31^ involved 2 multicentric case-control studies from central Europe (including Romania, Poland, and Russia) and Latin America (including Cuba, Argentina, and Brazil), while the other 8 were single center studies. All the cases under study were confirmed to have EC through histological, pathological or cytological means, and 6 articles of 7 studies were ESCC^17^,^27^,^29^,^31^,^32^,^33^. All the studies clearly defined the reference group of on the basis of tooth loss, with the major characteristics presented in Table 1. All the studies reported adjusted point estimates and 95% CIs. The adjusted covariates are shown in Table 2.

### Tooth loss and risk of EC

**Figure 2:**
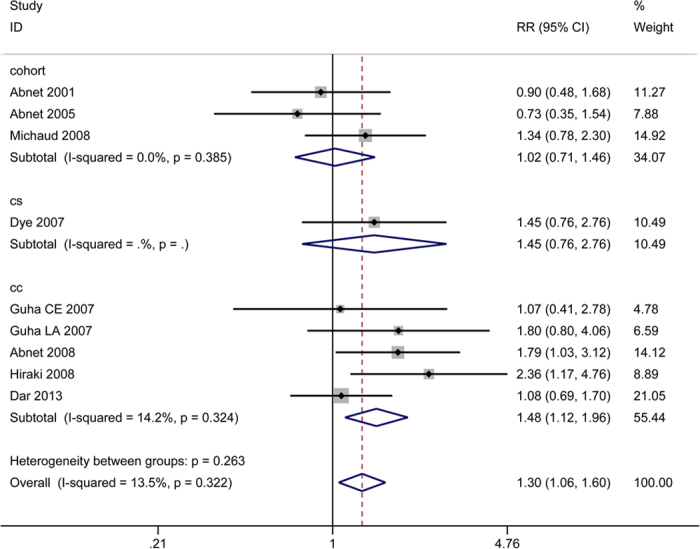
Forest plot of tooth loss and risk of esophageal cancer in overall population. Guha CE 2007, the study was conducted in Europe; Guha LA 2007, the study was conducted in Latin-America.

Among the eight included studies, two showed a significantly positive association between tooth loss and the risk of EC^29^,^30^, while the other six were negative^17^,^27^,^28^,^31^,^32^,^33^. No substantial heterogeneity among these trials was observed (*I*^2^ = 13.5%, *p* = 0.32), and the overall result based on the fixed effect model showed that tooth loss increased risk of EC by 1.30 times (RR = 1.30, 95%CI = 1.06–1.60; Fig. 2).

**Figure 3:**
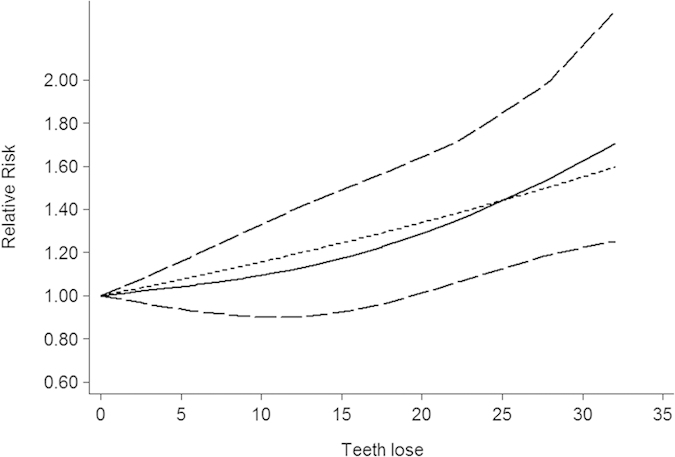
Dose-response analysis of every 1 tooth loss increment and risk of esophageal cancer. The black solid line and the black long dashed line represent the estimated RRs and corresponding 95% CIs for the non-linearity. The black short dashed line represents the estimated RRs for the linearity.

Five studies provided sufficient data of dose-response relationship. Our dose-repose meta-analysis showed an increased risk of EC corresponding to every 1 tooth loss increment (RR = 1.01, 95%CI = 1.00–1.03). No evidence of nonlinear relationship was detected (P = 0.45; Fig. 3).

Table 3 shows the results of subgroup analyses. The results of all subgroup analyses showed significantly increased risk in case-control studies and in adjustments for smoking and/or alcohol.

### Publication bias

**Figure 4:**
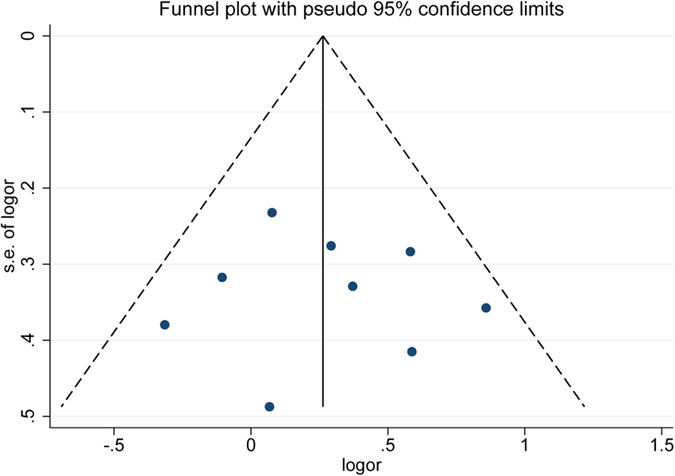
Funnel plot with pseudo-95% CIs of results of 5 studies based on the result of overall population.

Visual inspection of the funnel plot did not identify any substantial asymmetry (Fig. 4), which indicated no publication bias existed.

## Discussion

The present meta-analysis found evidence of an association between tooth loss and the risk of developing EC, with the loss of tooth significantly increasing the risk by 30%. And the relationship between tooth loss and the risk of developing EC is linearly dependent. Subgroup analyses revealed similar results of pooled estimations, and publication bias was not observed. In fact, this significant association could be explained. Periodontal disease is a chronic inflammatory disease, which contributing to constant low-grade systemic inflammation with elevated levels of circulating inflammatory markers^4^,^34^ and is associated with HNC^35^ and EC^36^,^37^. Periodontal disease is the major cause of tooth loss^38^,^39^, and tooth loss is also a maker of systemic inflammation^40^,^41^. The link between inflammation and cancer has long been recognized^42^,^43^,^44^,^45^, and it is likely that the progression of periodontal disease to tooth loss also represents a progression in the breakdown of normal cell-cycle control and potential carcinogenesis. In addition, the progression of tooth loss destroys the normal periodontal tissue, allowing oral microorganisms to accumulate deep into the oral tissue, thereby facilitating their growth. Oral microorganisms produce greater amounts of nitrosamine^46^, which is significantly associated with the development of cancer^47^,^48^. It is not difficult to envisage that during normal physiological behavior such as swallowing and drinking, oral microorganisms as well as the produced nitrosamine are passed into the esophagus from the oral cavity along with food and drinks; therefore, an association between tooth loss and EC seems plausible.

Subgroup analyses provided varied results of cross-sectional study design, which could be partly due to the smaller number of included studies or the limitations of this design; however, the pooled results from case-control studies and cohort studies presented a significantly increased risk of EC associated with tooth loss (Table 3). On the other hand, according to the Hill’s criteria, which are widely accepted for determining causality^49^, our meta-analysis indicates that tooth loss is a marker rather than a causal factor for EC. In our meta-analysis, all included studies did not show consistency in results, and pooled RRs only showed a weak association. In addition, only 3 cohort studies among the 10 included studies involved to temporality, although their pooled result showed a significant association. Furthermore, there is no evidence whether control of tooth loss can prevent EC up to now; therefore, the specificity of the association cannot be evaluated. Moreover, there is lack of experimental research. In summary, these factors suggest that that tooth loss is a marker rather than a causal factor for EC.

The heterogeneity was none to mild among the studies examining the association between tooth loss and the risk of EC^23^. This mild heterogeneity can be attributed to the differences in the characteristics of different populations, definitions of the reference and tooth loss group(s), and adjustment for confounding factors. Our subgroup analyses provide evidence that the study design, definition of the reference group, reported effect size, pathological type, and adjustment of origin was not the source of the heterogeneity (Table 3). Hence, the mild heterogeneity might be the inherent shortage of meta-analysis or due to the statistic heterogeneity.

Our meta-analysis also has some strengths and implications. To the best of our knowledge, this study is the first meta-analysis on this topic. We have searched relevant published studies via electronic and manual searching and collected all published studies that met the inclusion criteria; no publication bias has been detected. In addition, all included studies provided adjusted effect sizes. Nine studies have adjusted for smoking, while 7 have simultaneously adjusted for smoking and alcohol (Table 2). For smoking and alcohol are the well accepted risk factor of EC^9^,^10^,^11^,^12^,^13^,^14^,^15^,^16^, we conducted subgroup analysis to investigate the smoking adjusted studies and both smoking and alcohol adjusted studies, the results were both significantly and similar. Thereby ensuring the accuracy of the results obtained and indicating that tooth loss is independent of the conventional risk factors for EC. Furthermore, accumulating evidence and the large sample size of included studies provide the statistical power to present precise and reliable risk estimates relative to a single study. The results of the included studies are not consistent, and subgroup analysis revealed that the sample size could influence the result. Moreover, we could not identify a causal relationship between tooth loss and the risk of EC; the actions, such as toothbrushing^32^,^50^ are suggested to prevent tooth loss and this may decrease the risk of developing EC. Relevant animal studies and interventional studies are necessary to further explore the association between tooth loss and EC. Finally, people who cessation smoking, mild drinking, daily use of dental floss, and seeks periodontal therapy are all important ways of countering the increased risk of EC in these subjects.

Despite these strengths, several limitations should be acknowledged in our meta-analysis. First, the definition of the reference group and tooth loss used varied among studies. Nowadays, no international unification index for evaluating tooth loss in relevant studies is available, which could result in heterogeneity and increase the difficulty of performing meta-analysis, even resulting in failure to meet the criteria for meta-analysis. Second, substantial heterogeneity was observed among studies, although it was mild and very commonly found in meta-analysis of observational studies and is understandable, which cannot be ignored. We have investigated heterogeneity using subgroup analyses; however, the real source(s) of heterogeneity could not be identified. Third, subgroup analyses revealed that the results are significantly inconsistent with limited statistical power owing to a relatively small number of included studies. We used Hill’s criteria to argue causality and have discussed how the results could be influenced by the sample size; greater numbers of relevant studies addressing this topic are required for enlarging the sample size. Furthermore, while almost all included studies have adjusted for smoking and alcohol consumption, other unmeasured factors, such as diabetes, gastroesophageal reflux, and socioeconomic factors may confound the interpretation of an association between tooth loss and EC and potentially introduce bias. Only a few included studies have adjusted for these factors (Table 2). Fourth, although no evidence of publication bias was observed, the statistical power of our study is limited by a relatively small number of included studies; thus, it is difficult to be certain that there is no publication bias. These limitations of the present meta-analysis may affect the accuracy of our results. Finally, for lacking of relevant assessment tool of cross-sectional study, we could not assess the risk of bias of included studies^51^.

In summary, the present meta-analysis indicates that tooth loss is probably a significant and dependent risk factor for EC, suggesting that people who have lost teeth should pay attention to the symptoms and avoid other classical risk factors for EC. Moreover, according to the Hill’s criteria for causal inference, tooth loss is a marker rather than causality for EC.

## Additional Information

**How to cite this article**: Chen, Q.-L. *et al.* Tooth loss is associated with increased risk of esophageal cancer: evidence from a meta-analysis with dose-response analysis. *Sci. Rep.* **6**, 18900; doi: 10.1038/srep18900 (2016).

## Figures and Tables

**Table 1:** Characteristics of included studies in the meta-analysis.

References	Country	Study design	Sample sizes	Age (yrs)	Outcomes	Definition of reference group	Estimation (95%CI) (Highest vs. lowest)
Abnet 2001	China	Cohort	28868	57(12)*	ESCC	None lost tooth	0.90 (0.49–1.70)
Abnet 2005	Finland	Cohort	29124	57.2 ± 5.1^#^	ESCC	Lost ≤ 10 teeth	0.73 (0.35–1.55)
Dye 2007	China	CS	579	40–67	ESCC	Lost < 4 teeth	1.45 (0.76–2.76)
Guha 2007	Latin America	CC	173/1805	any age	ESCC	Lost ≤ 5 teeth	1.07 (0.41–2.77)
Guha 2007	Central Europe	CC	132/928	any age	ESCC	Lost ≤ 5 teeth	1.80 (1.80–4.07)
Michaud 2008	USA (White, Asian, Black)	Cohort	48375	40–75	EC	Lost ≤ 8 teeth	1.34 (0.78–2.30)
Abnet 2008	Iran	CC	283/560	65(56–73)/65(57–72)*	ESCC	Lost ≤ 12 teeth	1.79 (1.03–3.13)
Hiraki 2008	Japan	CC	354/708	20–79	EC	Lost ≤ 11 teeth	2.36 (1.17–4.75)
Dar 2013	India	CC	703/1664	61.6 ± 11.1/59.8 ± 11.1^#^	ESCC	None lost tooth	1.08 (0.68–1.69)

CS, cross-sectional; CC, case-control; EC, esophageal cancer; ESCC, esophageal squamous cell carcinoma;^*^, median (IQR);^#^, mean ± standard deviation.

**Table 2:** Adjustments in studies included in the meta-analysis

References	Adjustment
Abnet 2001	age, gender, smoking, and alcohol
Abnet 2005	age and education
Dye 2007	age, gender, village, education, smoking
Guha 2007	age, gender, country/center, education, smoking, alcohol, and all other oral health variables
Abnet 2008	age, gender, place of residence, ethnicity, alcohol, smoking, opium, or both, education, number of appliances, and fruit and vegetable intake
Hiraki 2008	age, gender, tobacco, alcohol, vegetable and fruit intake, body mass index, and regular exercise
Michaud 2008	age, race, physical activity, history of diabetes, alcohol, body-mass index, geographical location, height, calcium intake, total calorific intake, red-meat intake, fruit and vegetable intake, vitamin D score, and smoking
Dar 2013	age, ethnicity, residence, education, wealth score, fruit and vegetable intake, bidi smoking, gutka chewing, alcohol consumption and cumulative use of hookah, cigarette, and nass

**Table 3:** Results of subgroups analyses of pooled RRs and 95% CIs.

Subgroups	No. of studies	Heterogeneity		Effect model	Meta-analysis	
		*I*^2^(%)	*p*^*h*^		RR (95%CI)	*p*
Study design						
Cohort	3	0	0.39	Fixed	1.02 (0.71–1.46)	0.91
Case-control	5	14.2	0.32	Fixed	1.48 (1.12–1.96)	<0.01
Cross-sectional	1	NA	NA	NA	1.45 (0.76–2.76)	0.26
Outcomes						
ESCC	7	1.2	0.42	Fixed	1.21 (0.95–1.53)	0.13
Mixed	2	36.4	0.21	Fixed	1.66 (1.08–2.54)	0.02
Definition of reference group						
None lost tooth	2	0	0.64	Fixed	1.01 (0.70–1.46)	0.94
No. of lost tooth	7	6.4	0.38	Fixed	1.46 (1.14–1.89)	<0.01
Effect estimation						
Relative risk	1	NA	NA	NA	0.90 (0.48–1.68)	0.74
Hazard ratio	2	40.3	0.20	Fixed	1.09 (0.70–1.68)	0.71
Odds ratio	6	0	0.46	Fixed	1.47 (1.14–1.91)	<0.01
Adjustment						
Smoking	8	0	0.46	Fixed	1.37 (1.10–1.70)	<0.01
Smoking and alcohol	7	10.4	0.35	Fixed	1.36 (1.08–1.71)	0.01

No., number; ESCC, esophageal squamous cell carcinoma; RR, relative risk; CI, confidence interval.

## References

[b1] Adegboye A. R., Twetman S., Christensen L. B. & Heitmann B. L. Intake of dairy calcium and tooth loss among adult Danish men and women. Nutrition 28, 779–784 (2012). 22459555 10.1016/j.nut.2011.11.011

[b2] Gerritsen A. E., Allen P. F., Witter D. J., Bronkhorst E. M. & Creugers N. H. Tooth loss and oral health-related quality of life: a systematic review and meta-analysis. Health Qual Life Outcomes 8, 126 (2010). 21050499 10.1186/1477-7525-8-126 PMC2992503

[b3] Kisely S. *et al.* Advanced dental disease in people with severe mental illness: systematic review and meta-analysis. Br J Psychiatry 199, 187–193 (2011). 21881097 10.1192/bjp.bp.110.081695

[b4] Leng W. D., Zeng X. T., Kwong J. S. & Hua X. P. Periodontal disease and risk of coronary heart disease: An updated meta-analysis of prospective cohort studies. Int J Cardiol 201, 469–472 (2015). 26313869 10.1016/j.ijcard.2015.07.087

[b5] Watt R. G., Tsakos G., de Oliveira C. & Hamer M. Tooth loss and cardiovascular disease mortality risk–results from the Scottish Health Survey. PLoS One 7, e30797 (2012). 22363491 10.1371/journal.pone.0030797 PMC3282705

[b6] Desvarieux M. *et al.* Relationship between periodontal disease, tooth loss, and carotid artery plaque: the Oral Infections and Vascular Disease Epidemiology Study (INVEST). Stroke 34, 2120–2125 (2003). 12893951 10.1161/01.STR.0000085086.50957.22 PMC2677013

[b7] Zeng X. T. *et al.* Tooth loss and head and neck cancer: a meta-analysis of observational studies. PLoS One 8, e79074 (2013). 24260154 10.1371/journal.pone.0079074 PMC3829962

[b8] Zheng T. Z. *et al.* Dentition, oral hygiene, and risk of oral cancer: a case-control study in Beijing, People’s Republic of China. Cancer Causes Control 1, 235–241 (1990). 2102296 10.1007/BF00117475

[b9] Mathieu L. N., Kanarek N. F., Tsai H. L., Rudin C. M. & Brock M. V. Age and sex differences in the incidence of esophageal adenocarcinoma: results from the Surveillance, Epidemiology, and End Results (SEER) Registry (1973-2008). Dis Esophagus 27, 757–763 (2014). 24118313 10.1111/dote.12147 PMC3979505

[b10] Taylor G. W., Manz M. C. & Borgnakke W. S. Diabetes, periodontal diseases, dental caries, and tooth loss: a review of the literature. Compend Contin Educ Dent 25, 179–184, 186-178, 190; quiz 192 (2004). 15641324

[b11] Matthews J. C., You Z., Wadley V. G., Cushman M. & Howard G. The association between self-reported tooth loss and cognitive function in the REasons for Geographic And Racial Differences in Stroke study: an assessment of potential pathways. J Am Dent Assoc 142, 379–390 (2011). 21454843 10.14219/jada.archive.2011.0192 PMC3744362

[b12] Anand P. S., Kamath K. P., Shekar B. R. & Anil S. Relationship of smoking and smokeless tobacco use to tooth loss in a central Indian population. Oral Health Prev Dent 10, 243–252 (2012). 23094267

[b13] Heegaard K. *et al.* Amount and type of alcohol consumption and missing teeth among community-dwelling older adults: findings from the Copenhagen Oral Health Senior study. J Public Health Dent 71, 318–326 (2011). 22320290 10.1111/j.1752-7325.2011.00276.x

[b14] Zhang Y. Epidemiology of esophageal cancer. World J Gastroenterol 19, 5598–5606 (2013). 24039351 10.3748/wjg.v19.i34.5598 PMC3769895

[b15] Conway D. I. *et al.* Enhancing epidemiologic research on head and neck cancer: INHANCE - The international head and neck cancer epidemiology consortium. Oral Oncol 45, 743–746 (2009). 19442571 10.1016/j.oraloncology.2009.02.007

[b16] Mehanna H., Paleri V., West C. M. & Nutting C. Head and neck cancer–Part 1: Epidemiology, presentation, and prevention. BMJ 341, c4684 (2010). 20855405 10.1136/bmj.c4684

[b17] Abnet C. C. *et al.* Prospective study of tooth loss and incident esophageal and gastric cancers in China. Cancer Causes Control 12, 847–854 (2001). 11714113 10.1023/a:1012290009545

[b18] Moher D., Liberati A., Tetzlaff J., Altman D. G. & Group P. Preferred reporting items for systematic reviews and meta-analyses: the PRISMA statement. BMJ 339, b2535 (2009). 19622551 10.1136/bmj.b2535 PMC2714657

[b19] Ronksley P. E., Brien S. E., Turner B. J., Mukamal K. J. & Ghali W. A. Association of alcohol consumption with selected cardiovascular disease outcomes: a systematic review and meta-analysis. BMJ 342, d671 (2011). 21343207 10.1136/bmj.d671 PMC3043109

[b20] Robbins A. S., Chao S. Y. & Fonseca V. P. What’s the relative risk? A method to directly estimate risk ratios in cohort studies of common outcomes. Ann Epidemiol 12, 452–454 (2002). 12377421 10.1016/s1047-2797(01)00278-2

[b21] Bellocco R. *et al.* Alcohol drinking and risk of renal cell carcinoma: results of a meta-analysis. Ann Oncol 23, 2235–2244 (2012). 22398178 10.1093/annonc/mds022

[b22] Higgins J. P. & Thompson S. G. Quantifying heterogeneity in a meta-analysis. Stat Med 21, 1539–1558 (2002). 12111919 10.1002/sim.1186

[b23] Higgins J. P., Thompson S. G., Deeks J. J. & Altman D. G. Measuring inconsistency in meta-analyses. BMJ 327, 557–560 (2003). 12958120 10.1136/bmj.327.7414.557 PMC192859

[b24] Greenland S. Model-based estimation of relative risks and other epidemiologic measures in studies of common outcomes and in case-control studies. Am J Epidemiol 160, 301–305 (2004). 15286014 10.1093/aje/kwh221

[b25] Orsini N., Li R., Wolk A., Khudyakov P. & Spiegelman D. Meta-analysis for linear and nonlinear dose-response relations: examples, an evaluation of approximations, and software. Am J Epidemiol 175, 66–73 (2012). 22135359 10.1093/aje/kwr265 PMC3244608

[b26] Egger M., Davey Smith G., Schneider M. & Minder C. Bias in meta-analysis detected by a simple, graphical test. BMJ 315, 629–634 (1997). 9310563 10.1136/bmj.315.7109.629 PMC2127453

[b27] Abnet C. C. *et al.* Tooth loss is associated with increased risk of gastric non-cardia adenocarcinoma in a cohort of Finnish smokers. Scand J Gastroenterol 40, 681–687 (2005). 16036528 10.1080/00365520510015430

[b28] Michaud D. S., Liu Y., Meyer M., Giovannucci E. & Joshipura K. Periodontal disease, tooth loss, and cancer risk in male health professionals: a prospective cohort study. Lancet Oncol 9, 550–558 (2008). 18462995 10.1016/S1470-2045(08)70106-2 PMC2601530

[b29] Abnet C. C. *et al.* Tooth loss and lack of regular oral hygiene are associated with higher risk of esophageal squamous cell carcinoma. Cancer Epidemiol Biomarkers Prev 17, 3062–3068 (2008). 18990747 10.1158/1055-9965.EPI-08-0558 PMC2586052

[b30] Hiraki A., Matsuo K., Suzuki T., Kawase T. & Tajima K. Teeth loss and risk of cancer at 14 common sites in Japanese. Cancer Epidemiol Biomarkers Prev 17, 1222–1227 (2008). 18483345 10.1158/1055-9965.EPI-07-2761

[b31] Guha N. *et al.* Oral health and risk of squamous cell carcinoma of the head and neck and esophagus: results of two multicentric case-control studies. Am J Epidemiol 166, 1159–1173 (2007). 17761691 10.1093/aje/kwm193

[b32] Dar N. A. *et al.* Poor oral hygiene and risk of esophageal squamous cell carcinoma in Kashmir. Br J Cancer 109, 1367–1372 (2013). 23900216 10.1038/bjc.2013.437 PMC3778293

[b33] Dye B. A. *et al.* Using NHANES oral health examination protocols as part of an esophageal cancer screening study conducted in a high-risk region of China. BMC Oral Health 7, 10 (2007). 17640341 10.1186/1472-6831-7-10 PMC1993835

[b34] Moutsopoulos N. M. & Madianos P. N. Low-grade inflammation in chronic infectious diseases: paradigm of periodontal infections. Ann N Y Acad Sci 1088, 251–264 (2006). 17192571 10.1196/annals.1366.032

[b35] Zeng X. T. *et al.* Periodontal disease and risk of head and neck cancer: a meta-analysis of observational studies. PLoS One 8, e79017 (2013). 24194957 10.1371/journal.pone.0079017 PMC3806857

[b36] Divaris K. *et al.* Oral health and risk for head and neck squamous cell carcinoma: the Carolina Head and Neck Cancer Study. Cancer Causes Control 21, 567–575 (2010). 20049634 10.1007/s10552-009-9486-9 PMC2925153

[b37] Fitzpatrick S. G. & Katz J. The association between periodontal disease and cancer: a review of the literature. J Dent 38, 83–95 (2010). 19895866 10.1016/j.jdent.2009.10.007

[b38] Houshmand M. *et al.* Refining definitions of periodontal disease and caries for prediction models of incident tooth loss. J Clin Periodontol 39, 635–644 (2012). 22612722 10.1111/j.1600-051X.2012.01892.x

[b39] Upadhyaya C. & Humagain M. The pattern of tooth loss due to dental caries and periodontal disease among patients attending dental department (OPD), Dhulikhel Hospital, Kathmandu University Teaching Hospital (KUTH), Nepal. Kathmandu Univ Med J (KUMJ) 7, 59–62 (2009). 19483455 10.3126/kumj.v7i1.1767

[b40] Barros S. P., Suruki R., Loewy Z. G., Beck J. D. & Offenbacher S. A cohort study of the impact of tooth loss and periodontal disease on respiratory events among COPD subjects: modulatory role of systemic biomarkers of inflammation. PLoS One 8, e68592 (2013). 23950871 10.1371/journal.pone.0068592 PMC3738507

[b41] Buchwald S. *et al.* Tooth loss and periodontitis by socio-economic status and inflammation in a longitudinal population-based study. J Clin Periodontol 40, 203–211 (2013). 23379538 10.1111/jcpe.12056

[b42] van Kempen L. C., de Visser K. E. & Coussens L. M. Inflammation, proteases and cancer. Eur J Cancer 42, 728–734 (2006). 16524717 10.1016/j.ejca.2006.01.004

[b43] Tan T. T. & Coussens L. M. Humoral immunity, inflammation and cancer. Curr Opin Immunol 19, 209–216 (2007). 17276050 10.1016/j.coi.2007.01.001

[b44] Coussens L. M. & Werb Z. Inflammation and cancer. Nature 420, 860–867 (2002). 12490959 10.1038/nature01322 PMC2803035

[b45] Karin M., Lawrence T. & Nizet V. Innate immunity gone awry: linking microbial infections to chronic inflammation and cancer. Cell 124, 823–835 (2006). 16497591 10.1016/j.cell.2006.02.016

[b46] Abnet C. C. *et al.* Tooth loss is associated with increased risk of total death and death from upper gastrointestinal cancer, heart disease, and stroke in a Chinese population-based cohort. Int J Epidemiol 34, 467–474 (2005). 15659476 10.1093/ije/dyh375

[b47] Magee P. N. Nitrosamines and human cancer: introduction and overview. Eur J Cancer Prev 5 Suppl 1, 7–10 (1996). 8972286 10.1097/00008469-199609001-00002

[b48] Straif K. *et al.* Exposure to high concentrations of nitrosamines and cancer mortality among a cohort of rubber workers. Occup Environ Med 57, 180–187 (2000). 10810100 10.1136/oem.57.3.180 PMC1739921

[b49] Bird A. The epistemological function of Hill’s criteria. Prev Med 53, 242–245 (2011). 21843547 10.1016/j.ypmed.2011.07.009

[b50] Zeng X. T. *et al.* Meta-analysis on the association between toothbrushing and head and neck cancer. Oral Oncol 51, 446–451 (2015). 25753558 10.1016/j.oraloncology.2015.02.095

[b51] Zeng X. *et al.* The methodological quality assessment tools for preclinical and clinical studies, systematic review and meta-analysis, and clinical practice guideline: a systematic review. J Evid Based Med 8, 2–10 (2015). 25594108 10.1111/jebm.12141

